# Development of nanobodies targeting hepatocellular carcinoma and application of nanobody-based CAR-T technology

**DOI:** 10.1186/s12967-024-05159-x

**Published:** 2024-04-12

**Authors:** Keming Lin, Baijin Xia, Xuemei Wang, Xin He, Mo Zhou, Yingtong Lin, Yidan Qiao, Rong Li, Qier Chen, Yuzhuang Li, Jinzhu Feng, Tao Chen, Cancan Chen, Xinyu Li, Hui Zhang, Lijuan Lu, Bingfeng Liu, Xu Zhang

**Affiliations:** 1 Institute of Human Virology, Key Laboratory of Tropical Disease Control of Ministry of Education, Guangdong Engineering Research Center for Antimicrobial Agent and Immunotechnology, Zhongshan School of Medicine, Sun Yat-sen University, No. 74 Zhongshan Road 2, Yuexiu District, Guangzhou, Guangdong 510080 People’s Republic of China; 2 Department of Pathology, The First Affiliated Hospital, Sun Yat-sen University, Guangzhou, Guangdong People’s Republic of China; 3 Shenzhen Key Laboratory of Systems Medicine for Inflammatory Diseases, School of Medicine, Shenzhen Campus of Sun Yat-sen University, Shenzhen, Guangdong People’s Republic of China; 4 Department of Medical Oncology, The Third Affiliated Hospital of Sun Yat-sen University, No. 600 Tianhe Avenue, Guangzhou, Guangdong 510630 People’s Republic of China

**Keywords:** HCC, Nanoparticle, Nanobody, Phage display, Nb-derived CAR-T cell therapy

## Abstract

**Background:**

Chimeric antigen receptor T (CAR-T) cell therapy, as an emerging anti-tumor treatment, has garnered extensive attention in the study of targeted therapy of multiple tumor-associated antigens in hepatocellular carcinoma (HCC). However, the suppressive microenvironment and individual heterogeneity results in downregulation of these antigens in certain patients’ cancer cells. Therefore, optimizing CAR-T cell therapy for HCC is imperative.

**Methods:**

In this study, we administered FGFR4-ferritin (FGFR4-HPF) nanoparticles to the alpaca and constructed a phage library of nanobodies (Nbs) derived from alpaca, following which we screened for Nbs targeting FGFR4. Then, we conducted the functional validation of Nbs. Furthermore, we developed Nb-derived CAR-T cells and evaluated their anti-tumor ability against HCC through in vitro and in vivo validation.

**Results:**

Our findings demonstrated that we successfully obtained high specificity and high affinity Nbs targeting FGFR4 after screening. And the specificity of Nbs targeting FGFR4 was markedly superior to their binding to other members of the FGFR family proteins. Furthermore, the Nb-derived CAR-T cells, targeting FGFR4, exhibited significantly enhanced anti-tumor efficacy in both experiments when in vitro and in vivo.

**Conclusions:**

In summary, the results of this study suggest that the CAR-T cells derived from high specificity and high affinity Nbs, targeting FGFR4, exhibited significantly enhanced anti-tumor efficacy in vitro and in vivo. This is an exploration of FGFR4 in the field of Nb-derived CAR-T cell therapy for HCC, holding promise for enhancing safety and effectiveness in the clinical treatment of HCC in the future.

**Supplementary Information:**

The online version contains supplementary material available at 10.1186/s12967-024-05159-x.

## Introduction

Liver cancer, also known as liver malignancy, can be divided into two categories: primary and secondary. In the year 2020, primary liver cancer held the unfortunate position of being the sixth most common cancer with the third-highest mortality rate on a global scale [1]. Primary liver cancer mainly includes HCC, intrahepatic cholangiocarcinoma, and other special types. Remarkably, HCC constitutes more than 80% of all primary liver cancer cases worldwide [2].

Currently, the prognosis of HCC is discouraging, with a 5-year survival rate of less than 20% across different countries around the world [3, 4]. The treatments for HCC mainly include surgical treatment, transcatheter arterial chemoembolization (TACE) [5], radiotherapy, chemotherapy, molecular targeted drugs. However, these treatments generally have limitations. For instance, in the case of advanced patients, the primary treatments include TACE and oral administration of molecular targeted drugs. Nevertheless, these approaches prove effective in less than one-third of patients and provide only moderate increase in overall survival rate [6]. Therefore, the quest for optimizing treatment for HCC remains a worthwhile endeavor.

In 1989, Eshhar et al. first proposed the concept of CAR-T cells [7]. CAR-T cells recognize antigens through an antigen presentation mechanism independent of major histocompatibility complex. CAR-T cells could target tumor cells that express specific antigens, eliciting a series of T cell related responses, including cell proliferation, cytokine secretion, and cytotoxicity. Currently, a large number of studies dedicated to the treatment of HCC using CAR-T cells, achieving promising therapeutic outcomes in preclinical studies and clinical trials, primarily targeting antigens like GPC3, MUC-1, CEA [8]. However, the suppressive microenvironment within the tumor and individual heterogeneity pose challenges, leading to variations in the expression of the currently studied target antigens among different patients [9–11]. Consequently, it is crucial to explore additional potential therapeutic targets to refine anti-tumor treatment.

The FGF-FGFR pathway comprises of 22 human Fibroblast growth factors (FGFs) and four highly conserved transmembrane receptors (Fibroblast growth factor receptor 1–4, FGFR1–4) that possess an intracellular tyrosine kinase domain [12–14]. In addition, FGFR5(FGFRL1) has the ability to bind FGFs but lacks the tyrosine kinase domain. FGFR4 selectively binds to FGF19 and plays a role in regulating bile balance [15]. However, in the context of disease, FGFR4 is closely associated with the development of tumors. FGFR4 has been observed to be overexpressed in HCC cells [16, 17]. It binds to FGF19, promoting the proliferation of HCC cells while inhibiting their apoptosis Furthermore, FGFR4–FGF19 signaling axis also plays a role in the epithelial-mesenchymal transition of HCC [18]. Therefore, FGFR4 is a promising target for HCC treatment and is expected to become a target for CAR-T cell therapy for HCC.

In 1993, Hamers-Casterman et al. identified a natural antibody that exclusively consisted of heavy chains in camels [19]. The single variable domain with antigen-binding ability, found within these heavy chain antibodies, is referred to as a Nb. Nbs offer several advantages, including low molecular weight, high affinity, minimal aggregation, excellent stability, low immunogenicity. Consequently, they hold distinct advantages over traditional antibodies in the field of medical applications. Nbs have found widespread use in the diagnosis, tracing, and treatment of various disease, including tumor therapy [20–22]. Nb-derived CAR-T cells utilize the advantages of Nb and exhibit anti-tumor efficacy [23]. For instance, “Cilta-cel” was approved to treat multiple myeloma, marking it as the world’s first Nb-derived CAR-T cell therapy to be approved [24, 25]. In addition to the approved Nb-derived CAR-T cell therapy, several investigations are currently underway, targeting various antigens with Nb-derived CAR-T cell. For instance, the Nb-derived CAR-T cells targeting CD33 [26] have demonstrated robust T cell activation, cytokine production, and cytotoxicity. The bispecific Nb-derived CAR-T cells targeting CD13 and TIM3 have shown effective in eliminating tumor cells [27]. Additionally, the Nb-derived CAR-T cells targeting PD-L1 [28] have proven effective in eliminating liver cancer cells. Therefore, in this study, our aim was to develop of a specific Nb-derived CAR-T cell targeting FGFR4 to enhance therapeutic efficacy in treating HCC.

## Material and methods

### Cell lines

HEK293T, Huh7 and BXPC3 cell lines were obtained from American Type Culture Collection (ATCC) and cultured in Dulbecco’s modified Eagle medium (Gibco) supplemented with 10% fetal bovine serum (Gibco). HEK293F cell line was obtained from ATCC and cultured in Union-293 medium (Union Biotech). All media contained 1% penicillin–streptomycin (Gibco). All cells were cultured in humidified atmosphere containing 5% CO_2_ at 37 °C. Cell lines were confirmed that they were not contaminated by mycoplasma through testing for mycoplasma DNA.

### Western blot

Expression of FGFR4-His, anti-FGFR4 Nb-Fc and CAR moiety were detected by western blot assay after transfection of encoding vector to HEK293T cells. After 2 days, the supernatant was collected and the transfected cells were lysed using NP40 buffer containing protease inhibitors (Sigma). The supernatant or lysate was boiled after adding to SDS loading buffer. The proteins were separated using 4–12% SurePAGE (Genscript) and transferred to the nitrocellulose transfer membrane for immunoblotting. Expression of FGFR4-His was detected by anti-His tag antibody (Proteintech, 66005-1-Ig). Expression of CAR moiety was detected by anti-Flag tag antibody (MBL, M185-3). Other antibodies we used included anti-GAPDH antibody (Cat No. 10494-1-AP, Proteintech), goat anti-rabbit IRDye 800CW (Cat No. 926-32211, Li-cor), and goat anti-mouse IRDye 680RD (Cat No. 926-68070, Li-cor). Images were acquired by using the two-color infrared laser imaging system Odyssey (Li-cor) and analyzed through using Image Studio Lite Ver 4.0 software (Li-cor).

### Protein expression and purification

The sequence of recombination FGFR4 was inserted into pcDNA3.1 vector which was transfected into HEK293F cells for expression. The medium supernatant was incubated with Ni–NTA agarose (Cytiva). Proteins were eluted using Tris-NaCl buffer containing imidazole. The sequences of Nb-Fc were inserted into the pcDNA3.1 vectors which were transfected into HEK293T cells for expression. The medium supernatant was purified using protein A (Cytiva).The concentration of proteins was measured using BCA protein assay kit (Thermo).

### Immunization of alpaca and construction of electroporation bacteria library

A healthy alpaca received immunizations at 2-week intervals, with 500 μg of FGFR4-HPF nanoparticles dissolved in PBS and mixed with freunds adjuvant (F5881-10ML, F5506-10ML, Sigma) administered near the cervical lymph node each time, for a total of four immunizations. The peripheral blood was collected both before and after immunization for peripheral blood mononuclear cells (PBMCs) isolation and serum ELISA, followed by total RNA extraction. Subsequently, cDNA was synthesized using the PrimeScript reverse transcription reagent kit (TaKaRa, Osaka, Japan).The sequences of Nbs were amplified and inserted into the pComb3XSS vector. Then recombinant plasmids were electroporated into *E. coli* SS320 electrocompetent cells. Subsequently, electro-transfected library was infected with M13K07 helper phages to generate Nb-displaying phages library.

### Serum ELISA

The FGFR4 proteins were immobilized on 96-well microtiter plates and blocked with 3% BSA after washing with PBS. Plates were incubated with serums at various dilution gradients after washing with PBST. Subsequently, plates were incubated with goat anti-llama IgG antibody (HRP) (Abcam, ab112786) after washing with PBST. Finally, plates were added 100 μl TMB solution (Thermo) after washing with PBST and then added 100 μl stop solution (Solarbio). Absorption of plates was measured at 450 nm.

### Screening of Nbs and monoclonal phages ELISA

The Nbs targeting FGFR4 were screened using phage display technology. The phages were incubated with antigen. After incubation, the non-bound phages were washed using PBS and then the antigen-bound phages were eluted using trypsin. Subsequently, we amplified the eluted phages. The titers of phages were calculated after screening and amplification. The monoclonal phages were identified after 2 rounds of screening.

The FGFR4 proteins were immobilized on 96-well microtiter plates and then blocked with 3% BSA. The plates were incubated with different monoclonal phages and then incubated with mouse anti-M13 monoclonal antibody (HRP) (Sino Biological). The subsequent ELISA procedures are as described above.

### Flow cytometry

Cells were stained using antibody with the volume of 5 μL per million cells in 100 μL staining volume. Mouse anti-human PE-FGFR4 antibody (Abcam, 4FR6D3) was used for detection of FGFR4. The phages binding to Huh7 cells were detected with mouse anti-PE His antibody (Biolegend, 362603). Mouse anti-human FITC-CD3 antibody (Biolegend, 981002) was used for staining to detect T-cells in tumor tissues. Samples were detected by FACS Aria II (BD Biosciences) and analyzed using FlowJo software (Tree Star, USA).

### Molecular docking

The protein structure prediction website (Protein Homology/analogY Recognition Engine V 2.0) and AlphaFold Protein Structure Database were used to predict the structures of FGFR4 antigen’s extracellular domain proteins and Nbs. Subsequently, binding complex between the antigen and Nb was predicted using ZDOCK molecular docking website. Finally, the results of molecular docking were analyzed using PDBePISA website.

### Antibodies-antigen binding ELISA

For the evaluation of binding ability of Nbs, FGFR4 proteins were immobilized on 96-well microtiter plates. Subsequently, plates were incubated with different Nb-Fc after blocking. After washing, plates were incubated with goat anti-human IgG-Fc secondary antibody (HRP) (Sino Biological). The subsequent ELISA procedures are as described above.For the evaluation of specificity of Nbs, Nb-Fc were immobilized on 96-well microtiter plates. Subsequently, plates were incubated with different FGFR-His proteins after blocking. After washing, plates were incubated with mouse anti-His tag secondary antibody (HRP) (Ray antibody, China). The subsequent ELISA procedures are as described above.

### Surface plasmon resonance (SPR)

The interaction between antigen and antibody was evaluated using BIAcore T100 (Cytiva). The CM5 censor chip (Cytiva) was activated and then injected with FGFR4-His protein for immobilization using amine coupling kit (GE Healthcare). Subsequently, chip was blocked using ethanolamine HCl. The interaction status of antigen to antibody were monitored and recorded at serial concentrations. The equilibrium dissociation constant (KD) was calculation by the association constant (Ka) and dissociation constant (Kd).

### Construction of CAR lentiviral vectors

The transmembrane domain and intracellular structural domain of CAR molecule in lentiviral plasmid used in this study is consistent with previous studies in our laboratory [29, 30]. The intracellular structural domain contains CD28, 4-1BB (CD137) and CD3ζ. The Nb was ligated to CAR molecule to constructed the Nb-derived CAR moiety. The CAR moiety was inserted into the pHR lentiviral plasmid which constructed in our laboratory.

### Isolation of primary human T lymphocytes and transduction of recombinant lentiviral particles

PBMCs were derived from anonymous blood specimens obtained from healthy volunteers (Guangzhou Blood Center) using Ficoll-Hypaque gradient separation. T cells were purified using negative-selected magnetic beads (BD Biosciences) and cultured in KBM581 medium (Corning). T cells were activated with anti-CD3 and anti-CD28 antibody (2 μg/ml, Biolegend) and expanded with human IL-2 (10 ng/ml, R&D Systems). Lentiviral plasmids encoding CAR moieties, pMD.2G plasmids and psPAX2 plasmids were co-transfected into HEK293T cells using the PEI transfection system (Polysciences). Pseudotyped lentiviral supernatant was filtered with 0.45 μm membrane after 48 h. T cells were transduced with pseudoviruses under the condition of 8 μg/mL polybrene (Sigma) after activation and centrifuged at 350 × g at 37 ℃ for 90 min. After incubating at 37 °C for 8–10 h, pseudoviruses was removed and T cells were expanded in 6-well plate (Corning) at 37 ℃ using fresh medium with hIL-2 as described above. The T cells infected by pseudotyped lentiviruses can be detected by flow cytometry. In brief, the T cells infected by lentiviruses encoding CAR-Flag-IRES-GFP were detected by GFP. The T cells infected by lentiviruses encoding CAR-Flag-P2A-tCD19 (truncated human CD19) were detected by using anti-human PE-CD19 antibody (Biolegend, 302208). And the T cells transduced with empty lentivirus vector were used as the mock group (NC-T cells).

### Cytotoxicity assay

The cytotoxicity of CAR-T cells towards tumor cells was evaluated at different effector-to-target ratios (E:T ratios from 10:1 to 0.625:1). For instance, at the 10:1 ratio, there were 10^5^ effector cells (transduced T cells) and 10^4^ target cells. The effector cells used in the cytotoxicity assay represent the transduced T lymphocytes as a whole. The cytotoxicity was evaluated after co-culturing effector cells and target cells in 150 μl KBM581 medium at 37 ℃ for 24 h in 96-well V-bottom plates using CytoTox 96^®^ Non-Radioactive Cytotoxicity Assay (Promega) according to manufacturer’s instructions. Absorbance of wells were measured at 490 nm and merged the absorbance values of wells containing effector cells alone and target cells alone as background, and subtracted them from the values of the co-cultures. Wells containing target cells alone were lysed with the lysis reagent for 30 min at 37 ℃ and the absorbance values was set as maximum control. Cytotoxicity was calculated as follow: Cytotoxicity (%) = (Experimental value–Effector spontaneous value–Target spontaneous value)/(Target maximum value–Target spontaneous value) × 100% [30].

### Cytokine ELISA and enzyme-linked immunosorbent spot (ELISpot) assay

CAR-T cells were co-cultured with Huh7 cells (10^4^) at 10:1 in 150 μl KBM581 medium at 37 ℃ for 24 h in 96-well V-bottom plates to detect cytokines using Human IFN-γ, TNF-α, and Granzyme B Precoated ELISA Kits (Dakewe, China) according to the manufacturer's instructions. In brief, the supernatants were collected after co-culturing and incubated within pre-coated wells for 2 h at room temperature. The biotinylated primary antibodies were incubated for 1 h after washing 3 times. The streptavidin-HRP reagents were incubated for 30 min after washing 3 times. After washing the plate and adding TMB reaction substrate for 5–10 min, stop solution was added and the absorbance of well was measured at 450 nm.

CAR-T cells were co-cultured with Huh7 cells (10^4^) at 10:1 in 150 μl KBM581 medium at 37 ℃ for 24 h in prepackaged plate to detect cytokines using Human IFN-γ Precoated ELISpot Kit (Dakewe) according to manufacturer's instructions. In brief, prepackaged plates were washed for 6 time after removing the cells from plates. The biotinylated IFN-γ antibodies were incubated for 1 h at 37 °C. The streptavidin-HRP reagents were incubated for 1 h after washing 6 times. The AEC buffer was incubated for 10–20 min in dark after washing 5 times. Eventually, prepackaged plate was washed and then scanned using the ImmunoSpot S6 scan reader (Cellular Technology Ltd) and the number of T cells expressing IFN-γ was calculated by using ImmunoSpot software (Version 5.1.34) (Cellular Technology Ltd).

### Xenograft mouse model

We chose NCG mice (NOD/ShiLtJGpt-Prkdc^em26Cd52^Il2rg^em26Cd22^/Gpt, GuangDong GemPharmatech Co., Ltd.) to assess the anti-tumor efficacy in vivo. All mice experiments are ethical and approved by the Institutional Animal Care and Use Committee of Sun Yat-sen University. 2 × 10^6^ Huh7 cells (in 100 μl PBS) were subcutaneously (s.c.) inoculated into the right flank of 6–8-week-old female mice using the 1 ml disposable sterilized syringe with 0.45*16 RWLB size of needle (DOUBLE-DOVE). Tumor volume (V = long diameter × short diameter^2^/2) was calculated. 3 × 10^6^ CAR-T cells (in 200 μl PBS) were intravenously (i.v.) injected into mice when tumor volume reached 100 mm^3^. All mice were intraperitoneally (i.p.) injected with hIL-2 (1 μg per mouse) every 3 days.

We measured tumor diameter, mice weight and observed mice survival status every 3 days. In the survival analysis experiment, we have monitored the mice for long time and euthanized them at the ethical endpoint. The ethical endpoint of the experiment was defined as follows: the tumor volume exceeding 2000 mm^3^ at the final monitoring, or weakness observed in mice which include significant impairment of normal physiological activities in mice due to tumor growth or tumor rupture. In the experiment of monitor the tumor volume, we have monitored the tumor volume until the fifteenth day after CAR-T cells treatment. Mice were executed and then the major organs and tumor tissues were dissected for next evaluation. Part of tumor tissues were digested with collagenase type IV (Sigma). Cell suspensions were passed through 70 μm filters and tumor-infiltrating T cells were isolated using Human tumor infiltrating tissue mononuclear cell separation Kit (Solarbio).

### Hematoxylin–eosin (H&E) staining, immunohistochemistry (IHC) and immunofluorescence (IF)

We obtained the tumor tissues and major organs from euthanized mice. Organs included the heart, liver, spleen, lung and kidney. These samples were fixed, treated and stained according to standard procedures of Powerful Biology Co., Ltd (Powerful Biology, China).

### Statistical analysis

Statistical analyses were conducted using Graphpad Prism software. Data are presented as mean ± SEM. *P* < 0.05 was considered statistically significant.

## Results

### Construction of FGFR4 nanoparticles and immunization of alpaca

**Fig. 1:**
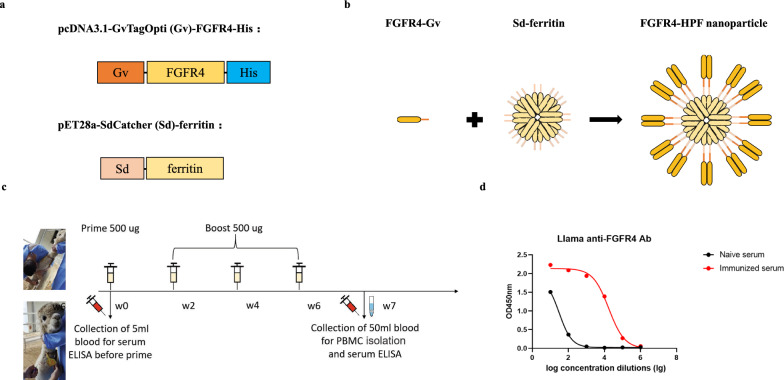
Construction of FGFR4 nanoparticles and immunization of alpaca. **a** Schematic diagram of expression vector encoding FGFR4 antigen’s extracellular domain proteins and ferritin. **b** Schematic diagram of generation of FGFR4-HPF nanoparticles. **c** Process of immunizing alpaca with FGFR4-HPF nanoparticles. **d** Immunized alpaca serum ELISA

In order to elicit more potent immune responses, we utilized the GvTagOpti/SdCatcher (Gv/Sd) system to present FGFR4 antigen on ferritin nanoparticles with higher efficiency [31–33]. We initiated the process by attaching a secretory signal peptide and Gv sequence at the N-terminus of the extracellular domain protein of FGFR4 antigen, while adding His tag to the C-terminus. Then, the Sd sequence was fused to the N-terminus of ferritin to achieve the expression of Sd-ferritin (Fig. 1a). Subsequently, we verified the expression of the Gv-FGFR4-His using western blot (Additional file 1: Fig. S1a). The next step involved covalently conjugating Gv-FGFR4-His to Sd-ferritin, resulting in the formation of FGFR4-HPF 24-mer nanoparticles (Fig. 1b). The presence of FGFR4-HPF was confirmed using coomassie blue staining (Additional file 1: Fig. S1b).

Subsequently we administered FGFR4-HPF nanoparticles to the alpaca in a total of four immunization rounds, while we collected the peripheral blood for serum ELISA (Fig. 1c). In the serum ELISA, we established specific criteria to determine positive antibody titers. Specifically, a serum sample was considered positive if the following conditions were met: (1) the absorbance of immunized serum was higher than that of naive serum, and (2) the absorbance of immunized serum should be greater than 0.5. These criteria help us identify and confirm the presence of specific antibodies in response to the immunization process.

The criteria for identifying the successful immune response in the alpaca included achieving positive antibody titers in the immunized serum within the range of 10^4^–10^5^. When compared with naive serum, the positive antibody titer in immunized serum was found to reach a dilution level of 10^4^ (Fig. 1d). This met the necessary conditions for proceeding with the subsequent library construction.

### Construction of electroporation bacteria library and screening of anti-FGFR4 Nbs

**Fig. 2:**
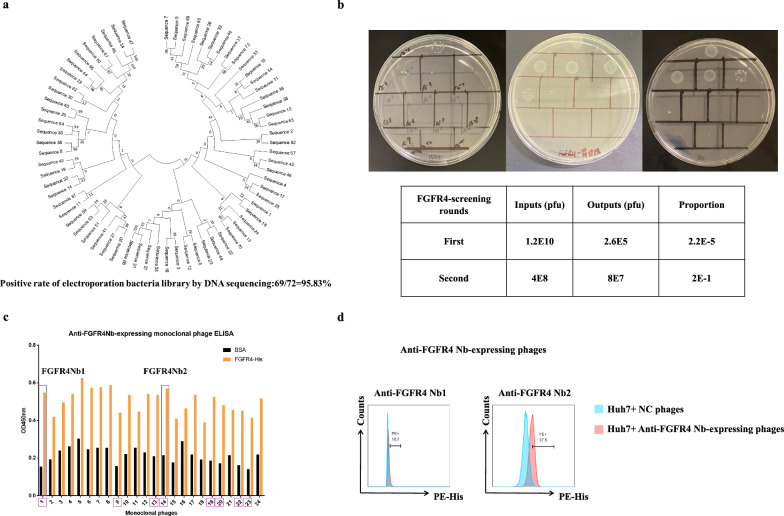
Construction of electroporation bacteria library and screening of anti-FGFR4 Nbs. **a** Monoclonal sequencing analysis and genetic evolutionary tree analysis of the electroporation library. Sequencing of 72 monoclonal clones identified 69 positive clones with insertion of single Nb sequences. **b** Proportion of Nb-displaying phages capable of binding to FGFR4 after screening. Left: output of first round; middle: input of second round; right: output of second round. **c** Monoclonal phage ELISA with FGFR4. Positive clones: 1, 9, 13, 14, 19. 20, 22, 23. **d** Identification of monoclonal phages capable of binding to Huh7 cells by flow cytometry. The NC phages uncapable of binding Huh7 cells were used as the control. Clones with positive fluorescence: 1 (anti-FGFR4 Nb1), 14 (anti-FGFR4 Nb2)

After completing immunization, we collected PBMCs from the alpaca. Subsequently, we amplified the sequences of the Nb and established the electroporation bacteria library. After calculating, the capacity of the electroporation library was 7.2 × 10^8^ and the clonal positivity rate of insertion was 93.75% (Additional file 1: Fig. S2a, b). Monoclonal sequencing further revealed that the positive rate of the electroporation library was 95.83% (Fig. 2a). Additionally, genetic evolutionary tree analysis demonstrated genetic diversity and non-repetitive amino acid sequences across all variable regions of Nbs. These further demonstrated the qualified sequence diversity within the electroporation library we had constructed. Next, we amplified the helper phage library and the initial phage library for screening Nbs targeting FGFR4 (Additional file 1: Fig. S2c). Following two rounds of screening, the titers of Nb-displaying phages capable of binding to FGFR4 were significantly enriched (Fig. 2b).

Subsequently, we conducted monoclonal phage ELISA to further assess the binding ability of the enriched phages. We considered an experimental well as positive when the ratio of the OD450 value of the experimental well to that of the control well (BSA-coated) was greater than or equal to 2.5. This criterion allowed us to identify a total of eight positive monoclonal clones (Fig. 2c). Furthermore, we conducted flow cytometry to verify these clones. In this process, we co-cultured these clones with Huh7 cells, which had been validated to overexpress FGFR4 (Additional file 1: Fig. S2d). Ultimately, we identified two monoclonal clones that exhibited positive fluorescence signals (Fig. 2d, Additional file 1: Fig. S2e).

### Functional validation of the identified Nbs in vitro

**Fig. 3:**
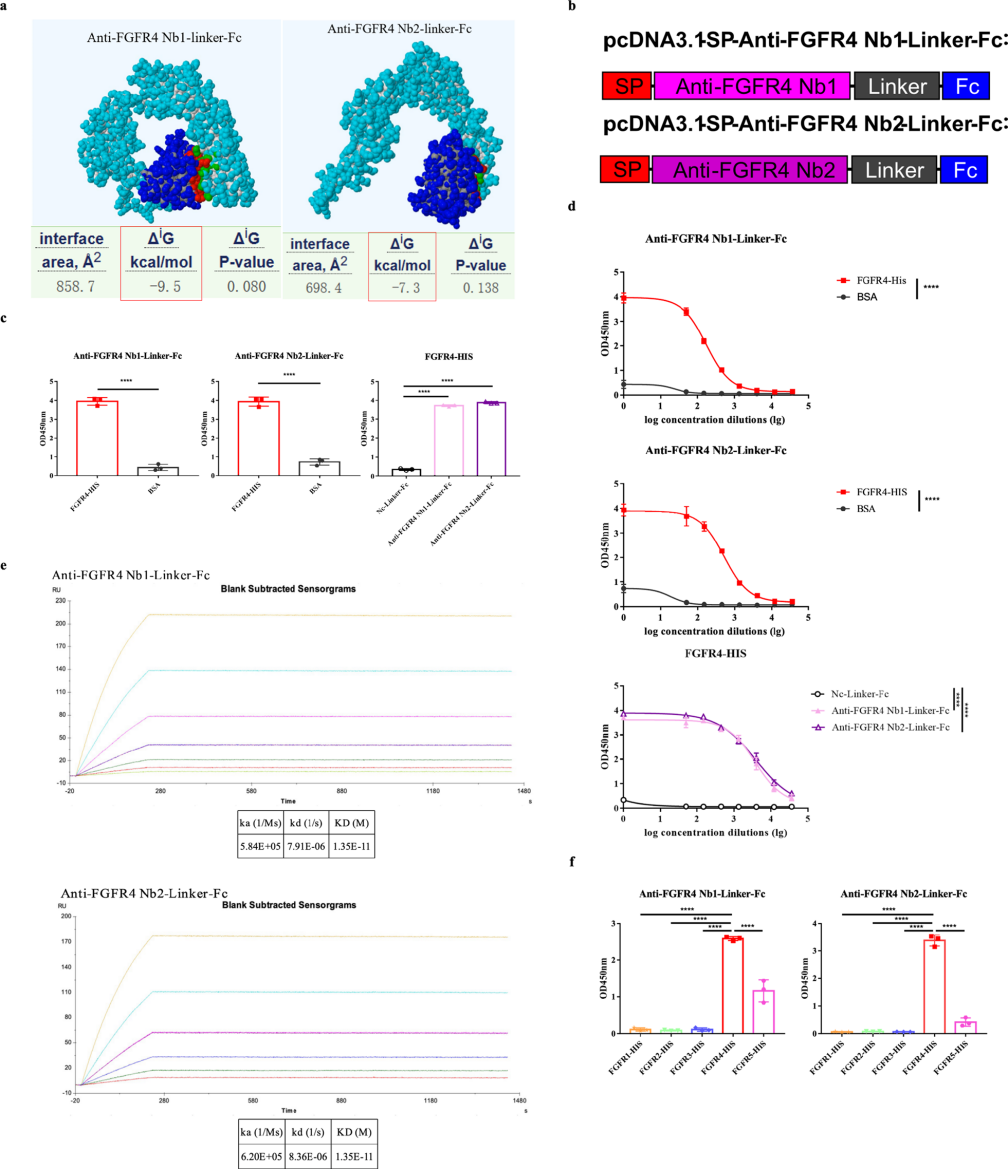
Functional validation of the screened Nbs in vitro*.* **a** Molecular docking models of Nbs with FGFR4. The structure of light blue represents antigen, the structure of dark blue represents Nbs, the interface regions of red and green represent the region of the Nb in contact with antigen, ΔG denotes the free energy of binding. **b** Schematic diagram of expression vector encoding Nb-Fc. **c** Detection of the binding ability of Nbs by antibody-antigen binding ELISA. Nc-Linker-Fc: non FGFR4 targeting control nanobody-Linker-Fc antibody. Data were analyzed by the Student’s t-test. **d** Detection of the binding ability of Nbs by antibody gradient dilution ELISA. Nc-Linker-Fc: non FGFR4 targeting control nanobody-Linker-Fc antibody. Data were analyzed by two-way ANOVA. **e** Detection of the binding affinity of Nbs by SPR assay. **f** Evaluation the specificity of Nbs by antibody-antigen binding ELISA. Data were analyzed by one-way ANOVA. The experiments were performed independently in triplicate. Data are expressed as mean ± SEM. ****p < 0.0001

Before conducting the functional validation of the Nbs, we conducted molecular docking to predict the binding ability between antigens and Nbs. Based on the results of the predictive model, the ΔG free energy of the Nbs is less than zero, it suggests their theoretical binding to the antigen (Fig. 3a).

Next, we conducted the functional validation of two Nbs. Firstly, we added a secretory signal peptide at the N-terminus of the Nbs and fused the human IgG Fc fragments at the C-terminus for construction of the Nb-Fc expression vectors (Fig. 3b). Then we verified their expression by western blot (Additional file 1: Fig. S3a). Subsequently, we purified Nb-Fc using protein A (Additional file 1: Fig. S3b).

To evaluate the binding ability of Nbs, we conducted antibody-antigen binding ELISA. The results indicated that the Nbs exhibited specificity and strong binding ability to FGFR4 compared with the BSA (Fig. 3c). Meanwhile, the results indicated that, compared with Nc-Linker-Fc antibody, anti-FGFR4 Nb-Linker-Fc antibody exhibited specificity and strong binding ability to FGFR4. Furthermore, we performed antibody gradient dilution ELISA. These experiments demonstrated the concentration-dependent binding of the Nbs to FGFR4, providing additional evidence of their binding abilities (Fig. 3d). For a more precise and quantitative assessment of affinity and kinetics between the Nbs and FGFR4, we conducted SPR experiments. The results were presented in the form of sensorgrams. Remarkably, the calculated KD values for both Nbs to FGFR4 can reach 1.35 × 10^–11^ (Fig. 3e).

To further evaluate the specificity of Nbs, we conducted ELISA using other FGFR family proteins. Based on the ELISA results, the Nbs displayed remarkable specificity and strong binding ability towards FGFR4. Importantly, this specificity was markedly superior to their binding to other FGFR proteins, including FGFR1-3 and FGFR5 (Fig. 3f). These results confirm the outstanding specificity and strong binding ability of the screened Nbs, particularly in their ability to target FGFR4. Consequently, we have successfully identified two Nbs that demonstrate remarkable specificity and strong binding ability for FGFR4, establishing a solid foundation for the construction of Nb-derived CAR-T cells targeting FGFR4.

### Construction of Nb-derived CAR-T cells and functional validation of the Nb-derived CAR-T cells anti-tumor in vitro

**Fig. 4:**
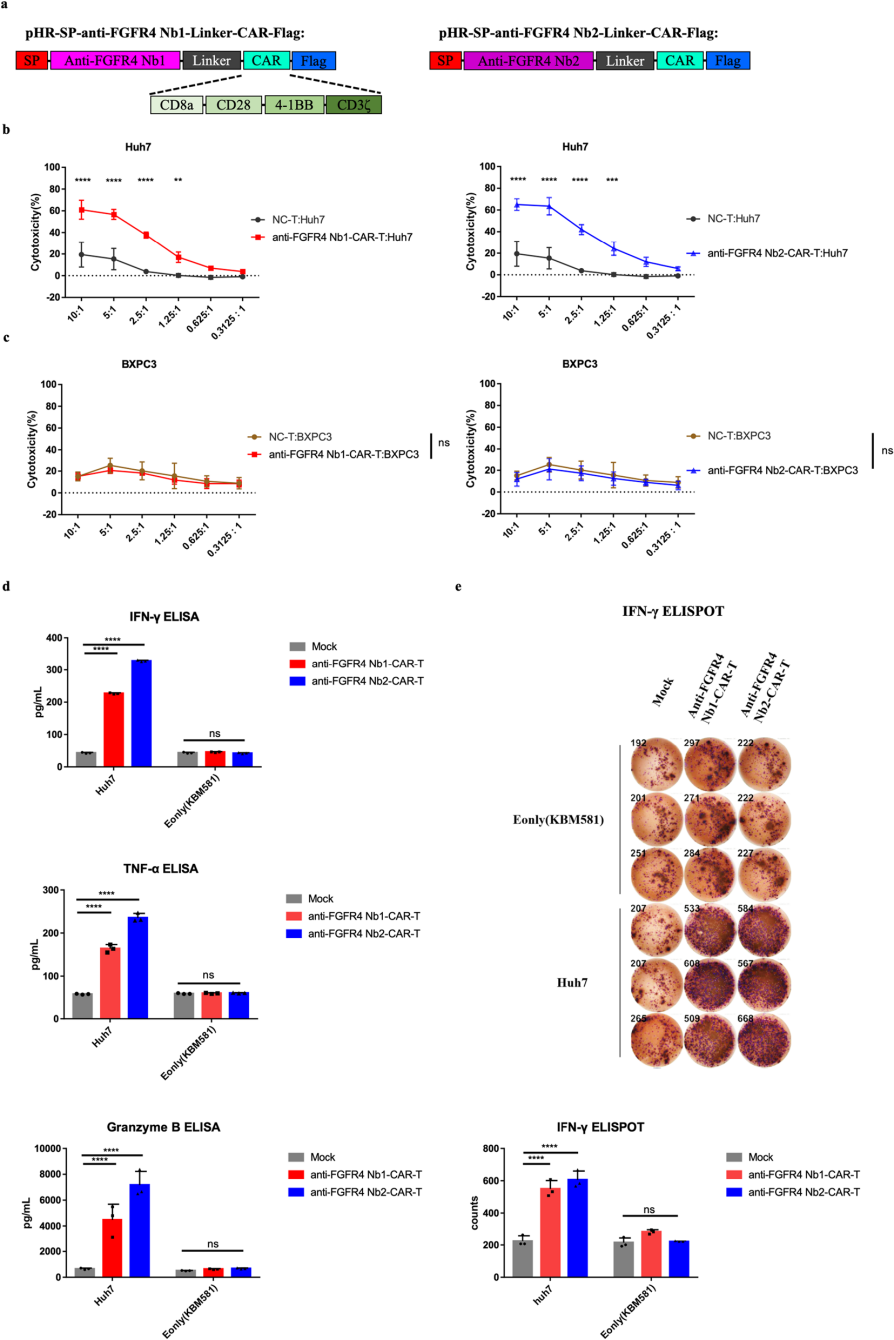
Construction of Nb-derived CAR-T cells and functional validation of Nb-derived CAR-T cells anti-tumor in vitro*.* **a** Schematic diagram of lentivirus plasmid encoding anti-FGFR4 Nb-CAR. **b** Validation of cytotoxicity of Nb-derived CAR-T cells against Huh7 cells in vitro by LDH assay. NC-T: T cells transduced with empty lentivirus vector. **c** Validation of cytotoxicity of Nb-derived CAR-T cells against BXPC3 cells in vitro by LDH assay. NC-T: T cells transduced with empty lentivirus vector. **d** Validation of the cytokine secretion functions of Nb-derived CAR-T cells against Huh7 cells in vitro by ELISA. Mock: T cells transduced with empty lentivirus vector. **e** Validation of the IFN-γ secretion functions of Nb-derived CAR-T cells against Huh7 cells in vitro by ELISPOT. Mock: T cells transduced with empty lentivirus vector. Data were analyzed by two-way ANOVA. The experiments were performed independent biological replicates (N = 3). Data are expressed as mean ± SEM. Ns: *p* > 0.05, **p < 0.01, ***p < 0.001, ****p < 0.0001

Based on the previous validation, we conducted functional validation by constructing Nb-derived CAR-T cells and evaluating their anti-tumor function in vitro in the next step. Firstly, we fused a secretory signal peptide at the N-terminus of the Nbs. Next, we added a CAR molecule and a Flag tag to the C-terminus to create the Nb-derived CAR moieties recombination lentiviral plasmids (Fig. 4a). Then we assessed the expression of CAR moieties via western blot (Additional file 1: Fig. S4a). Subsequently, T cells were transduced with pseudoviruses encoding the Nb-derived CAR moieties, and the expression of the CAR moieties was detected using flow cytometry after transduction (Additional file 1: Fig. S4b).To validate the in vitro anti-tumor function of Nb-derived CAR-T cells, Nb-derived CAR-T cells were co-cultured with Huh7 cells. In these experiments, both groups of Nb-derived CAR-T cells exhibited significant cytotoxicity against Huh7 cells when compared with the mock group across effector cell to target cell (E: T) ratios ranging from 10:1–1.25:1 (Fig. 4b).

Moreover, we validated the cytotoxic specificity by co-culturing Nb-derived CAR-T cells with BXPC3 cells. The BXPC3 cells was selected because it has been identified with extremely low expression of FGFR4 (Additional file 1: Fig. S4c). Notably, both groups of Nb-derived CAR-T cells displayed non-significant cytotoxicity against BXPC3 cells when compared with the mock group across all E:T ratios (Fig. 4c). As a result, it can be concluded that Nb-derived CAR-T cells effectively enhance anti-tumor function while maintaining specific cytotoxicity, as they specifically against cells with high expression of the FGFR4, such as Huh7 cells, without causing harm to cells with low or no FGFR4 expression, like BXPC3 cells.

Next, we assessed the in vitro effector cytokine secretion functions of Nb-derived CAR-T cells following co-culture with Huh7 cells. The levels of cytokines, including IFN-γ, TNF-α, and granzyme B, in the supernatants of Nb-derived CAR-T cells were found to be significantly higher than those secreted by the mock group after co-culturing with Huh7 cells (Fig. 4d). Furthermore, we also conducted an ELISpot assay to evaluate the secretion of IFN-γ in vitro. In line with the previous results, the levels of secreted IFN-γ in the supernatants of Nb-derived CAR-T cells were significantly higher than those secreted by the mock group after co-culturing with Huh7 cells (Fig. 4e). These findings provide additional validation of the anti-tumor function of Nb-derived CAR-T cells through their ability to secrete various effector cytokines.

### Functional validation of the Nb-derived CAR-T cells anti-tumor in vivo

**Fig. 5:**
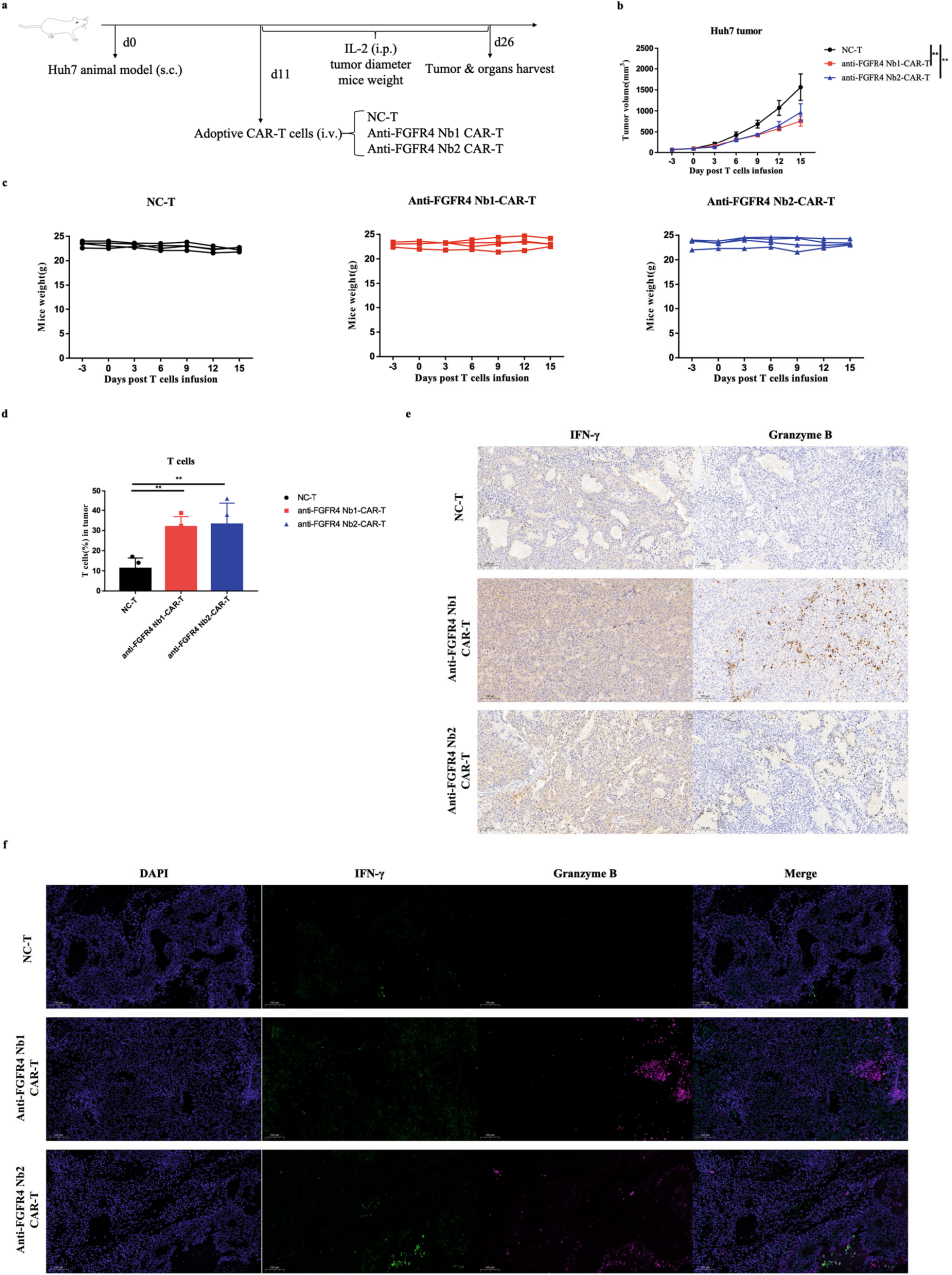

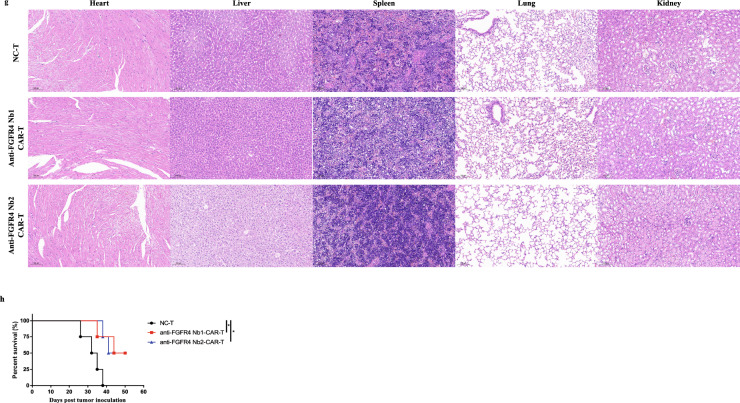
Functional validation of the Nb-derived CAR-T cells anti-tumor in vivo*.* **a** Schematic diagram of in vivo experiments on tumor-bearing mice. **b** Dynamic growth curve of tumor volume (*n* = 4). Data were analyzed by two-way ANOVA. NC-T: T cells transduced with empty lentivirus vector. **c** Dynamic change curve of mouse weight (*n* = 4). **d** Validation of the proportion of the tumor-infiltrating T cells by flow cytometry. Data were analyzed by one-way ANOVA. **e** IHC of the tumor tissue sections stained with anti-human IFN-γ (left) and granzyme B (right) antibody. Scale bar, 100 µm. **f** IF of the tumor tissue sections stained with anti-human IFN-γ (green) and anti-human granzyme B (purple) antibodies, and the nuclei were stained with DAPI (blue). Scale bar, 100 µm. **g** H&E staining of major organs. Scale bar, 100 µm. **h** Kaplan Meier survival analysis of mice (*n* = 4). Data were analyzed by log-rank Mantel-Cox test. Data are expressed as mean ± SEM. *p < 0.05, **p < 0.01

Based on the previous in vitro validation, we conducted in vivo anti-tumor functional validation in the next step. Nb-derived CAR-T cells were intravenously (i.v.) injected into immunodeficient mice that had been subcutaneously (s.c.) inoculated with Huh7 cells (Fig. 5a). The dynamic growth curve of tumors showed that the Nb-derived CAR-T cell group exhibited significant inhibition of tumor growth compared with the mock group (Fig. 5b, Additional file 1: Fig. S5a). Meanwhile, the dynamic change curve of weight demonstrated a non-significant fluctuation in the mice weight, indicating the safety of Nb-derived CAR-T cells (Fig. 5c).

After a period of 2–3 weeks following the infusion of Nb-derived CAR-T cells, the mice were euthanized. Then we dissected the tumor tissues for analyzing tumor-infiltrating T cells and their effector cytokine secretion function. Meanwhile, we dissected the major organs such as the heart, liver, spleen, lung, and kidney for assessing the potential toxicity.

Firstly, we assessed the tumor-infiltrating T cells using flow cytometry. The results revealed the significant increase of tumor-infiltrating T cells in the Nb-derived CAR-T cell groups compared with the mock group (Fig. 5d, Additional file 1: Fig. S5b). Secondly, we evaluated the cytokine secretion function of T cells within the tumor tissues using IHC and IF. The results indicated the significant increase in cytokine secretion levels in the Nb-derived CAR-T cell groups compared with the mock group, including IFN-γ and granzyme B (Fig. 5e, f). Furthermore, we assessed the condition of major organs using H&E staining. Images from H&E displayed that major organ shows no apparent inflammation, confirming the safety of Nb-derived CAR-T cells (Fig. 5g, Additional file 1: Fig. S5c). Additionally, we evaluated the in vivo therapeutic effect of Nb-derived CAR-T cells by monitoring the survival of tumor-bearing mice. The mice treated with Nb-derived CAR-T cells showed significantly improved survival compared with the mock group (Fig. 5h).

In conclusion, the results above demonstrate that Nb-derived CAR-T cells exhibit safety, superior anti-tumor functionality, and effective cytokine secretion in vivo, ultimately extending the lifespan of tumor-bearing mice.

## Discussion

In this study, we successfully screened and identified Nbs targeting FGFR4. Subsequently, we constructed Nb-derived CAR-T cells and evaluated their efficacy in treating HCC. The results demonstrated that the Nbs we confirmed targeting FGFR4 had high specificity and affinity. In particular, the specificity of Nbs targeting FGFR4 significantly outperformed their binding to other FGFR proteins. Moreover, the Nb-derived CAR-T cells we constructed exhibited the strong anti-tumor function in vitro and in vivo. This is an exploration of FGFR4 in the field of Nb-derived CAR-T cell therapy for HCC. Meanwhile, the Nb-derived CAR-T cell therapy we constructed is the first anti-FGFR4 Nb-derived CAR-T cell therapy for HCC.

HCC accounts for the majority of primary liver cancer cases globally. Unfortunately, the prognosis for HCC remains unfavorable. Currently, there are several approved drugs developed for HCC, encompassing tyrosine kinase inhibitors (TKIs), non-TKIs and monoclonal antibodies. Although these drugs diversify the treatment of HCC, they cannot meet the treatment requirements of all patients. For instance, sorafenib elicits a positive response in fewer than one-third of patients and often leads to drug resistance after several months of treatment [6]. CAR-T cell therapy functions as both a targeted drug and a “living drug”. Currently, there are multiple CAR-T cell therapies approved for treating hematological malignancies and achieved satisfactory outcomes [34–36]. Although CAR-T cell therapy for HCC is still in the clinical trial stage, clinical benefits have been observed in early clinical trials [37]. This suggests that CAR-T cell therapy holds significant promise for advancing the treatment of HCC.

However, owing to the tumor heterogeneity and the relatively limited number of identified targets for CAR-T cell therapy of HCC in both preclinical and clinical studies, the development of novel CAR-T cell targets specifically designed for HCC, which are currently unexplored in clinical trials, holds significant potential to diversify CAR-T cell therapy targets for HCC and increases the likelihood of approval of CAR-T cell therapy for HCC. Despite FGFR4 being a highly expressed antigen on HCC cells and the considerable advancements in development of FGFR4 inhibitors, there are still two challenges that need resolution. First, similar to many TKIs, FGFR4 inhibitors face issues related to drug resistance [38]. Second, currently reported FGFR4 inhibitors have not yet received approval. Thus, development of anti-FGFR4 CAR-T cells not only broadens the spectrum of CAR-T cell therapy targets for HCC but also compensates for the shortcomings of FGFR4 inhibitors.

Currently, CAR-T cell therapy predominantly employs single-chain variable fragment (scFv) as recognition domains. However, scFv exhibit issues related to aggregation [39–41] and immunogenicity [26, 42, 43], potentially impacting its therapeutic effectiveness. Conversely, Nbs have shown superior performance, and Nb-derived CAR-T cell hold promise in mitigating these limitations. Clinical data from approved Nb-derived CAR-T cell therapies indeed support the feasibility of developing CAR-T cells using Nbs [44–46]. Therefore, the anti-FGFR4 CAR-T cell therapy we developed uses self-developed Nbs. Based on our laboratory’s previous research [31, 32], the utilization of nanoparticle-based immunization has demonstrated enhancement in immune responses. Moreover, the Gv/Sd connection system [33] has demonstrated that it can enhance conjugation efficiency of the immunogen to nanoparticles. Therefore, we have chosen to depart from the conventional approach of immunizing alpacas with monovalent antigen, instead opting for nanoparticles presenting antigens using Gv/Sd system for alpaca immunization. This innovative approach is anticipated to elicit more robust immune responses and generate superior antibodies for subsequent screening. Evidently, our results have demonstrated that the Nbs we obtained by this approach had high specificity and affinity. Furthermore, the Nb-derived CAR-T cells, which we constructed using these Nbs, exhibit favorable therapeutic efficacy in vitro and in vivo.

In summary, our study has provided initial evidence supporting the feasibility of employing anti-FGFR4 Nb-derived CAR-T cell therapy in HCC. Furthermore, this strategy requires the establishment of patient-derived xenograft (PDX) model to further evaluate its cytotoxicity. If the PDX model demonstrates favorable therapeutic efficacy, the safety and effectiveness of this strategy are worthy of further validation in clinical trials. Our study offers promise for developing novel Nb-derived CAR-T cell therapy for HCC.

## Conclusions

In this study, we employed a novel approach by utilizing covalently linked antigen nanoparticles for alpaca immunization, aiming to identify highly specific and high-affinity Nbs through screening. Expanding on this foundation, we successfully engineered the first Nb-derived CAR-T cells targeting FGFR4 for HCC. This pioneering exploration of FGFR4 within Nb-derived CAR-T cell therapy for HCC holds promise for enhancing both the safety and efficacy of future clinical treatments for HCC.

### Supplementary Information

**Additional file 1: Figure S1.** Purification and identification of the FGFR4 antigen’s extracellular domain proteins and FGFR4-HPF proteins.** a** Identification of the expression vector encoding FGFR4 proteins by western blot. **b** Identification of the FGFR4-HPF proteins by coomassie blue staining. **Figure S2**. Construction of electroporation bacteria library and screening of anti-FGFR4 Nbs. **a** Calculation of the capacity of the electroporation library by counting the number of clones on the plate after gradient dilution. **b **Identification of Nb gene clonal positivity rate of insertion by colony PCR. M: 5000 bp DNA marker. **c** Calculation of the capacity of the helper phage library and the initial phage library after amplification. **d** Identification of FGFR4 expression of Huh7 cell line by flow cytometry. FGFR4 expression of Huh7 cell line was shown through gating on PE + population. **e**: Identification of monoclonal phages capable of binding to Huh7 cells by flow cytometry. The monoclonal phages capable of binding Huh7 cells was shown through gating on PE + population. The NC phages uncapable of binding Huh7 cells were used as the control. **Figure S3. **Purification and identification of the Nb-Fc. **a** Identification of the expression vector encoding Nb-Fc by western blot. **b** Identification of the Nb-Fc after purification by coomassie blue staining. **Figure S4. **Detection of Nb-derived CAR and FGFR4 expression. **a** Identification of the lentivirus plasmid expressing anti-FGFR4 Nb-CAR by western blot. **b** Detection of the expression of the CAR moieties by flow cytometry. CAR expression of T cells was shown through gating on FITC + population (GFP) or PE + population (tCD19). NC-T: T cells transduced with empty lentivirus vector. **c** Identification of FGFR4 expression of BXPC3 cell line by flow cytometry. FGFR4 expression of BXPC3 cell line was shown through gating on PE + population. **Figure S5**. Functional validation of the Nb**-**derived CAR-T cells anti-tumor in vivo. **a** Dynamic growth curve of tumor volume (*n* = 3). NC-T: T cells transduced with empty lentivirus vector. **b** Validation of the proportion of the tumor-infiltrating T cells by flow cytometry. The proportion of the tumor-infiltrating T cells was shown through gating on CD3 + population. **c** An enlarged view of the H&E staining of the main organs in Fig. 5g. Scale bar, 50 µm

## Data Availability

The datasets used and/or analysed during the current study are available from the corresponding author on reasonable request.

## Abbreviations

CAR-T: Chimeric antigen receptor T
HCC: Hepatocellular carcinoma
HPF: Ferritin
Nbs: Nanobodies
TACE: Transcatheter arterial chemoembolization
FGFs: Fibroblast growth factors
FGFR: Fibroblast growth factor receptor
ATCC: American Type Culture Collection
Gv/Sd: GvTagOpti/SdCatcher
PBMC: Peripheral blood mononuclear cells
SPR: Surface plasmon resonance
KD: Equilibrium dissociation constant
Ka: Association constant
Kd: Dissociation constant
ELISA: Enzyme-linked immunosorbent assay
ELISpot: Enzyme-linked immunosorbent spot
H&E: Hematoxylin–eosin
IHC: Immunohistochemistry
IF: Immunofluorescence
TKIs: Tyrosine kinase inhibitors
scFv: Single-chain variable fragment
PDX: Patient-derived tumor xenograft
