# Supplementary material for: Development of nanobodies targeting hepatocellular carcinoma and application of nanobody-based CAR-T technology
Source: J Transl Med. 2024 Apr 12;22:349. doi: 10.1186/s12967-024-05159-x (PMC11015683; doi:10.1186/s12967-024-05159-x)
Supplement: Supplementary file 1 — Additional file 1: Figure S1. Purification and identification of the FGFR4 antigen’s extracellular domain proteins and FGFR4-HPF proteins. a Identification of the expression vector encoding FGFR4 proteins by western blot. b Identification of the FGFR4-HPF proteins by coomassie blue staining. Figure S2. Construction of electroporation bacteria library and screening of anti-FGFR4 Nbs. a Calculation of the capacity of the electroporation library by counting the number of clones on the plate after gradient dilution. b Identification of Nb gene clonal positivity rate of insertion by colony PCR. M: 5000 bp DNA marker. c Calculation of the capacity of the helper phage library and the initial phage library after amplification. d Identification of FGFR4 expression of Huh7 cell line by flow cytometry. FGFR4 expression of Huh7 cell line was shown through gating on PE + population. e: Identification of monoclonal phages capable of binding to Huh7 cells by flow cytometry. The monoclonal phages capable of binding Huh7 cells was shown through gating on PE + population. The NC phages uncapable of binding Huh7 cells were used as the control. Figure S3. Purification and identification of the Nb-Fc. a Identification of the expression vector encoding Nb-Fc by western blot. b Identification of the Nb-Fc after purification by coomassie blue staining. Figure S4. Detection of Nb-derived CAR and FGFR4 expression. a Identification of the lentivirus plasmid expressing anti-FGFR4 Nb-CAR by western blot. b Detection of the expression of the CAR moieties by flow cytometry. CAR expression of T cells was shown through gating on FITC + population (GFP) or PE + population (tCD19). NC-T: T cells transduced with empty lentivirus vector. c Identification of FGFR4 expression of BXPC3 cell line by flow cytometry. FGFR4 expression of BXPC3 cell line was shown through gating on PE + population. Figure S5. Functional validation of the Nb-derived CAR-T cells anti-tumor in vivo. a Dynamic growth curve of tumo [file 12967_2024_5159_MOESM1_ESM.pdf]

Figure S1

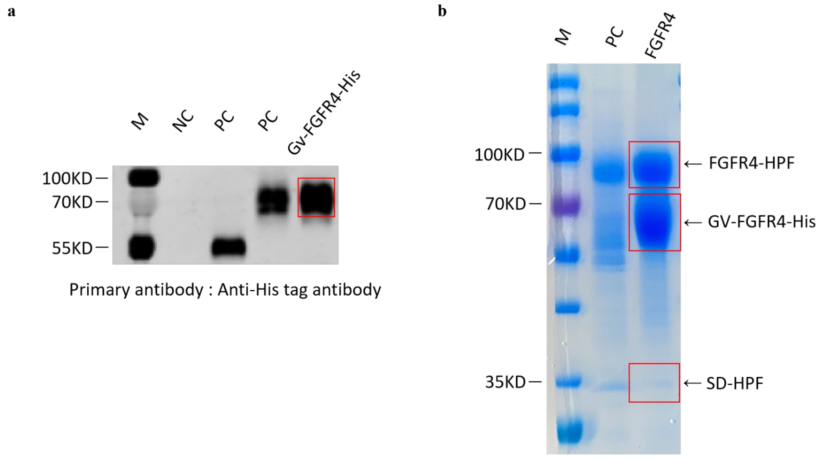
a
M
NC
PC
PC
Gv-FGFR4-His
100KD
70KD
55KD
Primary antibody : Anti-His tag antibody
b
M
PC
FGFR4
100KD
70KD
35KD
FGFR4-HPF
GV-FGFR4-His
SD-HPF

Figure S2

a

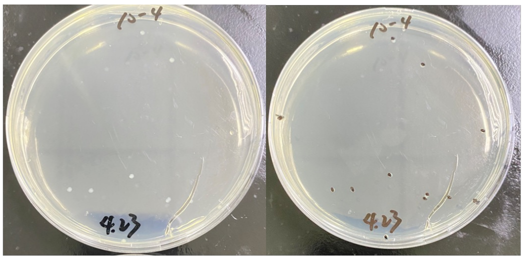

Capacity of electroporation bacteria library:12x10^{4}x10^{3}x6=7.2E8

b

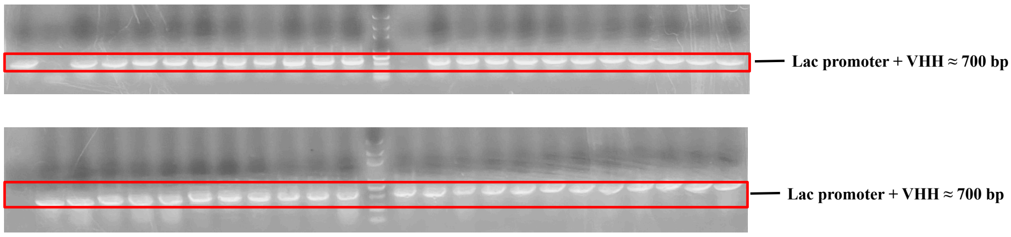

Positive rate of electroporation bacteria library by colony PCR : 45/48=93.75%

c

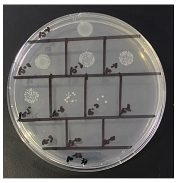

Capacity of initial phage library:3x400x10^7=1.2E10

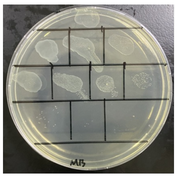

Capacity of helper phage library:（20+30）/2x400x10^9=1E13

d

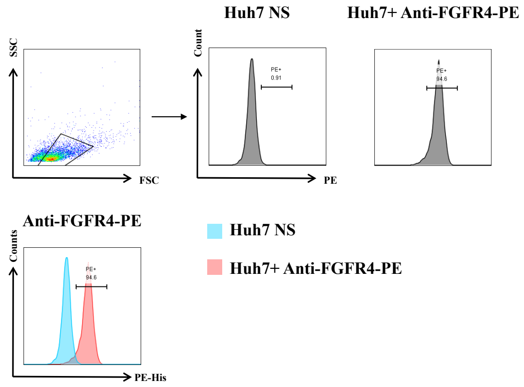

e

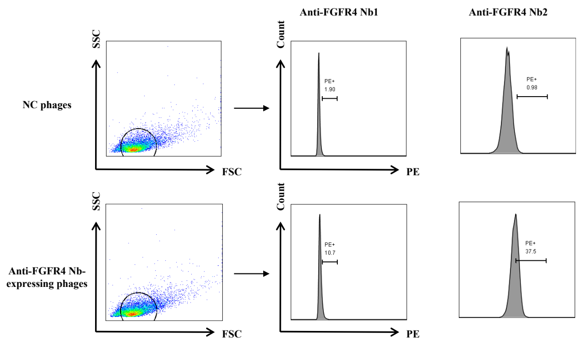

Figure S3

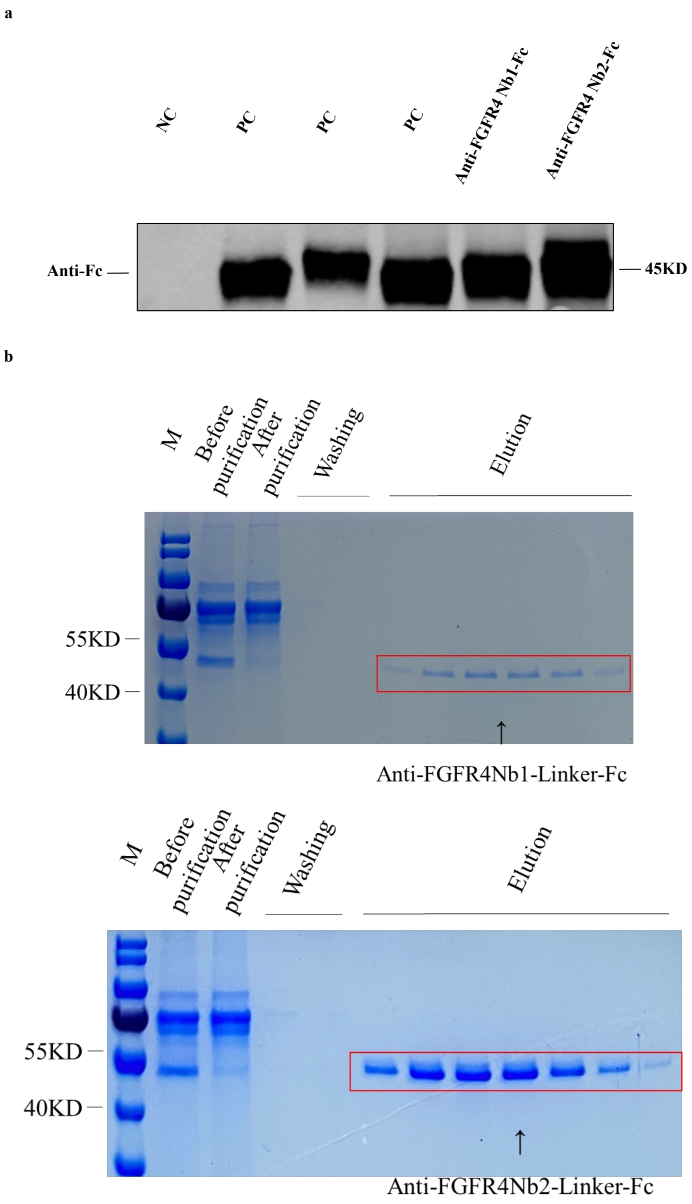
a
NC
PC
PC
PC
Anti-FGFR4 Nb1-Fc
Anti-FGFR4 Nb2-Fc
Anti-Fc
45KD
b
M
Before purifcation
After purifcation
Washing
Elution
55KD
40KD
Anti-FGFR4Nb1-Linker-Fc
M
Before purification
After purification
Washing
Elution
55KD
40KD
Anti-FGFR4Nb2-Linker-Fc

Figure S4

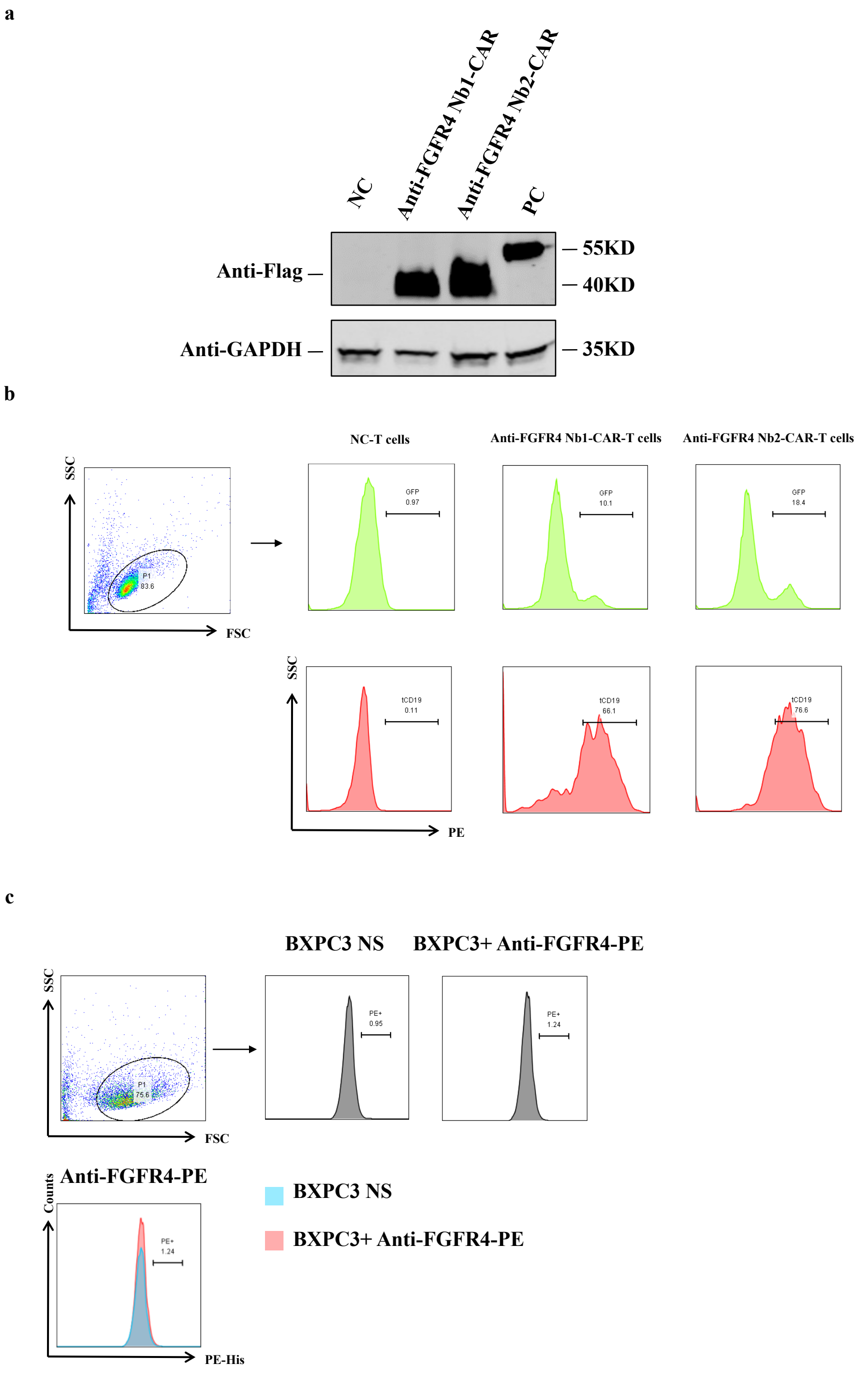
a
NC
Anti-FGFR4 Nb1-CAR
Anti-FGFR4 Nb2-CAR
PC
Anti-Flag
Anti-GAPDH
55KD
40KD
35KD
b
NC-T cells
Anti-FGFR4 Nb1-CAR-T cells
Anti-FGFR4 Nb2-CAR-T cells
SSC
FSC
P1
83.6
GFP
0.97
GFP
10.1
GFP
18.4
SSC
PE
tCD19
0.11
tCD19
66.1
tCD19
76.6
c
BXPC3 NS
BXPC3+ Anti-FGFR4-PE
SSC
FSC
P1
75.6
PE+
0.95
PE+
1.24
Anti-FGFR4-PE
Counts
PE+
1.24
PE-His
BXPC3 NS
BXPC3+ Anti-FGFR4-PE

Figure S5

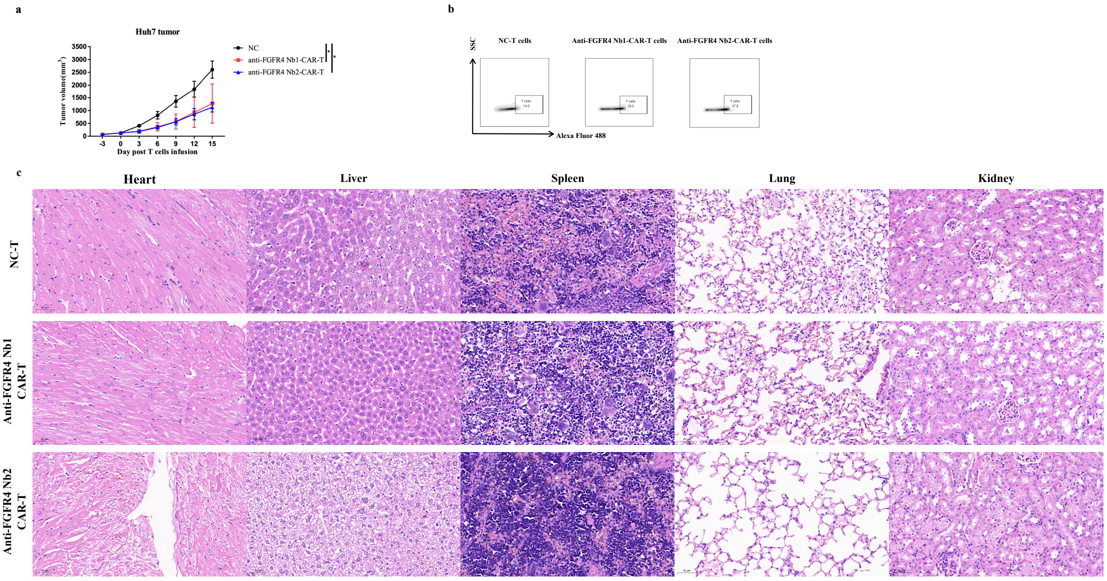
a
Huh7 tumor
Tumor volume(mm³)
3500
3000
2500
2000
1500
1000
500
0
-3
0
3
6
9
12
15
Day post T cells infusion
NC
anti-FGFR4 Nb1-CAR-T
anti-FGFR4 Nb2-CAR-T
*
*
b
SSC
NC-T cells
Anti-FGFR4 Nb1-CAR-T cells
Anti-FGFR4 Nb2-CAR-T cells
T cells
14.0
T cells
29.0
T cells
37.8
Alexa Fluor 488
c
Heart
Liver
Spleen
Lung
Kidney
NC-T
Anti-FGFR4 Nb1 CAR-T
Anti-FGFR4 Nb2 CAR-T
